# An Outbreak of *Clostridium difficile* Ribotype 027 Associated with Length of Stay in the Intensive Care Unit and Use of Selective Decontamination of the Digestive Tract: A Case Control Study

**DOI:** 10.1371/journal.pone.0160778

**Published:** 2016-08-17

**Authors:** Yvette H. van Beurden, Olaf M. Dekkers, Marije K. Bomers, Annie M. Kaiser, Robin van Houdt, Cornelis W. Knetsch, Armand R. J. Girbes, Chris J. J. Mulder, Christina M. J. E. Vandenbroucke-Grauls

**Affiliations:** 1 Department of Medical Microbiology and Infection Control, VU University medical center, Amsterdam, The Netherlands; 2 Department of Gastroenterology and Hepatology, VU University medical center, Amsterdam, The Netherlands; 3 Department of Clinical Epidemiology, Leiden University Medical Center, Leiden, The Netherlands; 4 Department of Endocrinology and Metabolic Diseases, Leiden University Medical Center, Leiden, The Netherlands; 5 Department of Internal Medicine, VU University medical center, Amsterdam, The Netherlands; 6 Department of Medical Microbiology, Leiden University Medical Center, Leiden, The Netherlands; 7 Department of Intensive Care, VU University medical center, Amsterdam, The Netherlands; Cleveland Clinic, UNITED STATES

## Abstract

**Background:**

An outbreak of *Clostridium difficile* ribotype 027 infection (CDI) occurred at an university hospital, involving 19 departments. To determine what hospital-associated factors drove the outbreak of this particular strain we performed a case-control study.

**Methods:**

Cases (n = 79), diagnosed with CDI due to *C*. *difficile* ribotype 027 were matched for age and treating medical specialty to four control patients (n = 316). Patients diagnosed with CDI due to other ribotypes were included as a second control group. A random selection of *C*. *difficile* ribotype 027 strains (n = 10) was genotyped by Whole Genome Sequencing (WGS).

**Findings:**

WGS showed the outbreak was likely caused by a single strain of *C*. *difficile* (two or less single-nucleotide variants between isolates). Ninety-five percent of cases had used antibiotics, compared to 56% of controls. Previous admission to the intensive care unit (ICU) (OR: 2.4, 95% CI 1.0–5.6), longer length of stay (LOS), and recent hospital admission were associated with CDI ribotype 027. Cases were less likely to have been admitted to a ward with a known isolated CDI patient (OR: 0.2, 95% CI 0.1–0.6).

Analysis of patients who stayed at the ICU (35 cases; 51 controls), indicated that the use of selective decontamination of the digestive tract (SDD) and a longer LOS in the ICU were associated with CDI risk.

**Interpretation:**

In this large outbreak, any antibiotic use, including SDD use, appeared as a prerequisite for acquisition of the outbreak strain. The role of use of SDD and prolonged stay on the ICU could not be disentangled, but both factors can play a biologically plausible role in *C*. *difficile* acquisition and infection.

## Introduction

The most frequent infectious cause of nosocomial diarrhea is *Clostridium difficile* [1]. The clinical manifestations of infection with toxin-producing strains of *C*. *difficile* range from mild or moderate diarrhea, to fulminant and sometimes fatal pseudomembranous colitis. The major cause of most outbreaks of *Clostridium difficile* infection (CDI) is *C*. *difficile* PCR ribotype 027: a more virulent ribotype. This ribotype has been associated with significantly higher morbidity and mortality, related to more severe complications [2,3]. The most important risk factor for CDI is antibiotic use; antibiotics from almost all classes have been associated with infection. Other well-described risk factors for CDI include ≥ 65 years of age, extensive comorbidity, and prolonged hospital stay [4–6].

Between May 2013 and July 2014 an outbreak of a single clone of *C*. *difficile* ribotype 027 occurred at the VU University medical center (VUmc), a 750-bed tertiary care center. It involved 19 departments and 79 patients. Several individual risk factors for CDI are known, but we hypothesized that, in addition, factors particular to hospital stay, like stay on a specific ward or undergoing a specific intervention, or multiple ward transfers may have increased the risk of the spread of the outbreak strain. Primary aim of this case-control study was to determine what hospital-associated factors increased the risk of developing CDI and therefore contributed to the hospital-wide spread of the outbreak strain.

## Material and Methods

### Ethics Statement

Approval for the study was obtained from the Medical Ethics Committee VUmc. We were exempted from obtaining written informed consent from subjects because of the retrospective design of our study. All collected data were anonymized.

### General study outline

In this case-control study we compared CDI patients infected with *C*. *difficile* ribotype 027, diagnosed between May 2013 and March 2014, with non-CDI controls, and controls with CDI due to other ribotypes for distribution of hospital-associated factors and clinical outcome parameters.

### Participants

All consecutive patients (n = 79) with CDI due to ribotype 027, hospitalized at adult wards in the specified time period, were included as cases. CDI was defined as the presence of diarrhea (≥3 unformed stools per 24 hours) in combination with positive stool culture for a *C*. *difficile* strain of ribotype 027. Patients diagnosed with CDI at the outpatient clinic were excluded.

For every case, four non-CDI control patients were included (n = 316). Controls were matched to cases on age, attending specialty, and stay at the ward within ±48 hours of the index date. The index date for cases was defined as the date of sampling of a *C*. *difficile* positive stool. Controls had no known history of CDI. A patient could be included as a control subject for only one case.

All consecutive patients diagnosed with CDI due to other ribotype strains (n = 70) between May 2013 and March 2014 were included as a second control group.

### Microbiological methods

We used the microbiological methods described by Van Prehn et al. [7]. In summary, stool samples of unformed fecal specimens were tested for toxin A and B with the VIDAS CDAB enzyme-linked fluorescence assay (Biomérieux, France) according to the manufacturer’s protocol. Samples were also cultured selectively under anaerobic conditions. Suspect colonies were confirmed to be *C*. *difficile* by Maldi-TOF mass spectrometry (Vitek MS, Biomérieux, France).

All isolates of *C*. *difficile* are routinely typed in our microbiology laboratory by Amplified Fragment Length Polymorphism (AFLP) [8]. AFLP permits differentiation between ribotypes 014, 017, 027, 078, 087, and other. A random selection of ribotype 027 isolates of the outbreak (n = 10; three from the beginning, three from the middle, and four from the end of the outbreak) was also characterized by Whole Genome Sequence (WGS) analysis. The DNA libraries were sequenced with the 150 bp paired-end chemistry on the Illumina MiSeq platform, according to the manufacture’s protocol [9].

### Data collection

Medical charts were reviewed for demographic and clinical characteristics. Hospital-associated characteristics (e.g. wards of stay, visits to diagnostic facilities) were also recorded. For every participant we collected the following data: age, gender, date and department of admission, date of discharge, date and department of CDI diagnosis, Charlson comorbidity index [10], LOS in the hospital prior to the index date, all wards of stay (prior to the index date), number of in-hospital transfers (prior to the index date), types and number of imaging sessions, and types and number of diagnostic procedures (e.g. endoscopy). Furthermore, data were collected on recent hospitalization, and in-hospital use of antibiotics and proton-pump inhibitors (PPI) for a period up to three months before the index date. For patients who stayed at the intensive care unit (ICU) we also collected data on Acute Physiology and Chronic Health Evaluation (APACHE IV) score [11], and use of selective decontamination of the digestive tract (SDD: intestinal and oropharyngeal application of colistin, tobramycin and amphotericin in combination with systemic cefotaxime during the first two to four days). Of note, all patients admitted at the ICU with an expected LOS of more than 48 hours receive SDD by protocol. Outcome measures were: mortality, total LOS, surgical intervention, admission to the ICU or medium care unit (MCU) after the index date, and CDI recurrence. Participants were followed for 90 days.

All relevant data can be found in the paper. For issues of patient privacy, individual patient data are available on request to the Ethics Committee of the VUmc for researchers who meet criteria to access confidential patient data.

### Statistical analysis

For 98% of patients clinical and demographical data were complete. Distribution of risk factors and clinical outcome parameters among patients with CDI due to ribotype 027 was compared to non-CDI control patients, and to patients with CDI due to other ribotypes. Odds ratios (OR) and their 95% confidence intervals (95% CI) were estimated by univariate and multivariable conditional logistic regression (STATA version 12.1). Factors associated in univariate analysis (p ≤ 0.20), as well as putative risk factors from earlier studies, were analyzed in the multivariable model. We did not adjust for age (matched variable). We performed log transformation for the variables hospital LOS and ICU LOS given their skewed distribution. The association between CDI and the use of antibiotics was analyzed using two different multivariable models. In the first model, a single class of antibiotics was analyzed by conditional logistic regression, adjusting for sex, Charlson’s index, hospital LOS, and prior admission to the ICU. In the second model, additional adjustment for the concomitant use of different classes of antibiotics was made by entering all classes of antibiotics that were associated with CDI in univariate analysis (p ≤ 0.20) into the multivariable model.

Factors that were significantly associated with CDI ribotype 027 in the multivariable model (complete 95% C.I. > 1.0) were analyzed in a separate multivariable model comparing cases with CDI ribotype 027 to control patients with CDI due to other ribotypes. The association between CDI ribotype 027, and a single class of antibiotics in this group was analyzed by logistic regression, adjusting for ICU admission, and hospital LOS.

In a second step we performed logistic regression restricted to cases and controls who had stayed at the ICU prior to the index date (the low number of cases and controls did not allow for a matched analysis). Use of SDD, hospital and ICU LOS prior to index date, number of ward transfers, and APACHE IV score were analyzed in the multivariable model.

AFLP patterns were analyzed with Bionumerics V6.6 [8]. Whole genome sequence data were analyzed by assembling paired-end reads with a *C*. *difficile* ribotype 027 reference genome, FN545816. Quality control and single-nucleotide variants (SNVs) analysis was performed as previously described by Knetsch et al [12].

## Results

### The outbreak

**Fig 1:**
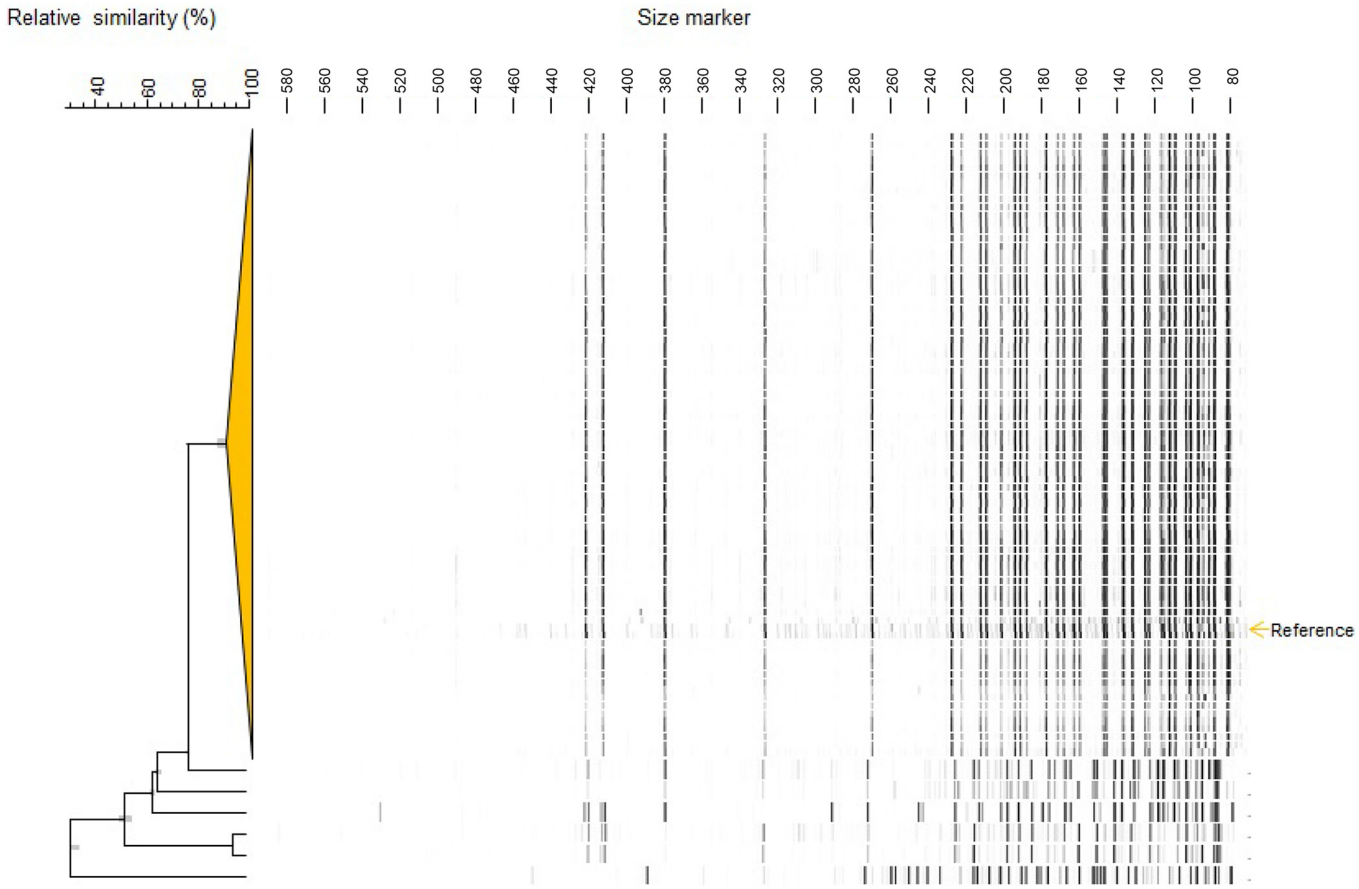
Dendrogram of *C*. *difficile* strains based on AFLP patterns of all ribotype 027 strains identified between May 2013 and March 2014. Hierarchical clustering of AFLP types was performed using unweighted pair-grouping. The cut-off value for identical strains was set at 90% relative similarity (BioNumerics, Applied Maths, Belgium). Besides 027 strains, a few other ribotypes that were found in the VUmc in the same period were included in the analysis, serving as an outgroup. All 027 strains included in this analysis were considered to be identical (large cluster on top).

**Fig 2:**
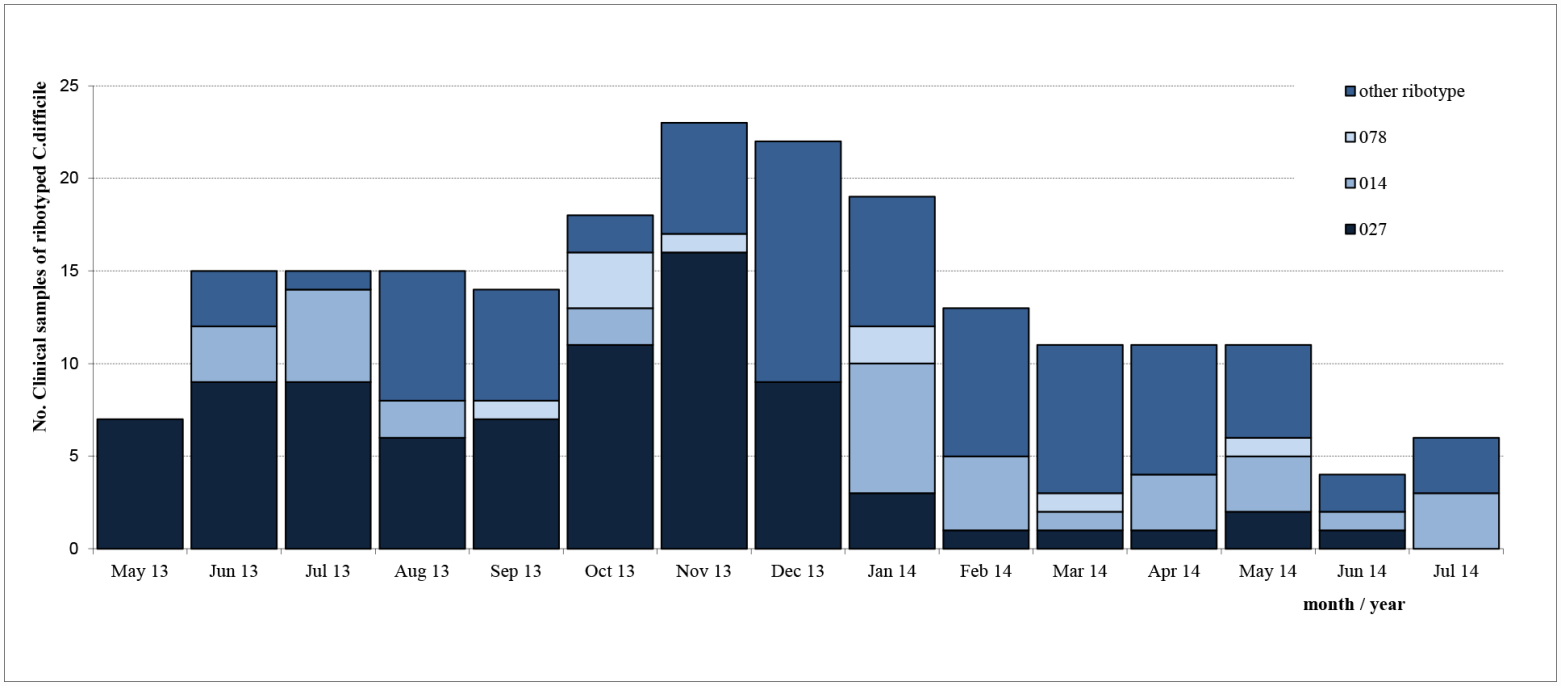
Monthly incidence of *C*. *difficile* infection from May 2013 to July 2014.

In May 2013 an increase of cases of CDI at the neurology ward was noticed. Typing of the *C*. *difficile* isolates showed that they were all of ribotype 027 (Fig 1). During the ensuing months *C*. *difficile* ribotype 027 spread to several wards, with an incidence of 50 cases per 10,000 admissions between May and December 2013. WGS of 10 strains of ribotype 027 isolated at different time points (May, June, September, October, November, December 2013, January 2014) during the outbreak, suggested that the cases of CDI were caused by clonally related *C*. *difficile* isolates (two or less SNVs between isolates). As shown in Fig 2, other ribotypes of *C*. *difficile* were also found during this period, but ribotype 027 was the most prominent. Most severely affected wards were: the combined department of vascular surgery, nephrology and urology, the department of neurology and the ‘transfer ward’, where patients no longer in need of hospital care can rehabilitate before discharge. However, the department of diagnosis could differ from the department of acquisition, since the time-interval between acquisition of *C*. *difficile* and developing CDI remains unknown. After the implementation of several control measures, the incidence of CDI due to ribotype 027 finally decreased to around 9 cases per 10,000 admissions in early 2014.

### Patient characteristics and risk factors for CDI

#### CDI due to ribotype 027 versus non-CDI controls

Patient characteristics are shown in Table 1. There were no large differences between case and controls in median age, sex, and Charlson’s index.

**Table 1:** Patient characteristics of patients with CDI and control patients.

Risk factors		CDI patients (N = 79)		Non-CDI control patients (N = 316)	
		N	(%)	N	(%)
Age (mean ±SD)		67	(±17)	67	(± 16)
Male		50	(63)	178	(57)
Charlson comorbidity index*					
	0	16	(20)	64	(20)
	1–2	34	(43)	156	(49)
	3–4	20	(25)	66	(21)
	≥ 5	9	(11)	29	(9)
Any antibiotic therapy (in 90 days)*		75	(95)	182	(58)
Antibiotic classes					
	Cephalosporins	62	(79)	95	(54)
	Cephalosporins 1^st^ generation	3	(4)	10	(3)
	Cephalosporins 2^nd^ and 3^rd^ generation without SDD	25	(32)	54	(17)
	Penicillins	38	(48)	82	(26)
	Quinolones	32	(41)	62	(20)
	Clindamycin	4	(5)	19	(6)
	Metronidazole	19	(24)	17	(5)
	SDD	38	(48)	49	(16)
	Vancomycin	15	(19)	23	(7)
	Carbapenems	10	(13)	27	(9)
	Aminoglycosides	10	(13)	31	(10)
	Macrolides	15	(19)	20	(6)
Proton pump inhibitors (-90 days)*		58	(73)	186	(59)
Number of ward transfers					
	0	20	(25)	140	(44)
	1–2	39	(49)	150	(48)
	≥ 3	20	(25)	26	(8)
Admission ICU		35	(44)	51	(16)
Length of stay before index date					
	≤ 5 days	14	(18)	129	(41)
	6–10 days	11	(14)	70	(22)
	11–25 days	28	(35)	67	(21)
	> 25 days	26	(33)	50	(16)
Recent hospital admission (-90 days)		27	(34)	78	(25)
Admission to the same ward as an isolated CDI patient		51	(65)	234	(74)

* Data was missing for one patient.

SDD: selective decontamination of the digestive tract; ICU: intensive care unit

In univariate analysis (Table 2) several factors were associated with CDI, including use of antibiotics and PPI, admission to the ICU, number of ward transfers, and LOS, all prior to index date. Cases were less likely to have been admitted to a ward with a known patient with CDI. Except for admission to the ICU, or to the same ward as an isolated CDI patient, none of the other hospital-associated factors we investigated was associated with spread of the outbreak strain. In multivariable analysis (Table 2), antibiotic use, LOS >10 days, prior admission to the hospital, and admission to the ICU prior to the index date remained significantly associated with the development of CDI; admission to the same ward as an isolated patient with CDI ribotype 027 remained a protective factor. After adjustment for sex, Charlson’s index, hospital LOS, and admission to the ICU, the use of SDD, cephalosporins 2^nd^ and 3^rd^ generation (without SDD), metronidazole, penicillins, and quinolones was associated with the development of CDI (Table 3, model 1). After adjustment for concomitant use of different classes of antibiotics, use of SDD, cephalosporins 2^nd^ and 3^rd^ generation (without SDD), and quinolones remained significantly associated with the development of CDI (Table 3, model 2). Additional adjustment for age (matched variable) did not materially change the results.

**Table 2:** Crude and adjusted odds ratios of potential risk factors for the development of CDI ribotype 027 compared to non-CDI control patients.

Risk factors		Crude OR (95% CI)	Adjusted OR^#^ (95% CI)
Age		1.00 (0.98–1.03)	NA
Male		1.35 (0.81–2.22)	1.03 (0.57–1.85)
Charlson comorbidity index		1.00 (0.98–1.01)	1.00 (0.98–1.02)
Any antibiotic therapy (-90 days)		14.10 (5.01–39.67)	5.67 (1.82–17.69)
Proton pump inhibitors (-90 days)		1.97 (1.12–3.47)	1.29 (0.65–2.59)
Number of transfers during prior to index date			
	0	Reference	Reference
	1–2	2.43 (1.21–4.88)	1.30 (0.54–3.15)
	≥ 3	7.16 (3.05–16.81)	1.38 (0.40–4.74)
Admission to ICU		6.00 (3.18–11.32)	2.36 (1.00–5.56)
Length of stay prior to index date			
	≤ 5 days	Reference	Reference
	6–10 days	1.85 (0.76–4.48)	2.30 (0.76–6.97)
	11–25 days	4.85 (2.18–10.78)	4.13 (1.42–12.02)
	≥ 25 days	7.91 (3.28–19.10)	4.83 (1.54–15.16)
Admission to the same ward as an isolated CDI patient		0.40 (0.19–0.86)	0.24 (0.10–0.62)
Previous admission (- 90 days)		1.63 (0.94–2.81)	1.86 (0.94–3.70)

The univariate and multivariable analysis were done by conditional logistic regression.

^#^ Model adjusted for sex, Charlson comorbidity index, antibiotic and PPI use, number of ward transfers, admission to ICU, length of stay, recent admission, and admission to the same ward as an isolated CDI patient. We did not adjust for the matched variable age.

ICU: intensive care unit; NA: not applicable

**Table 3:** Crude and adjusted odds ratios of antibiotic classes as a risk factor for CDI ribotype 027 compared to non-CDI control patients.

	Crude odds ratio (95% CI)	Adjusted OR (95% CI) model 1^#^	Adjusted OR (95% CI) model 2^##^
Cephalosporins 2^nd^ and 3^rd^ generation without SDD	2.31 (1.31–4.06)	3.97 (2.03–7.77)	5.03 (2.29–11.04)
Penicillins	2.69 (1.61–4.52)	1.82 (1.02–3.27)	1.30 (0.67–2.55)
Quinolones	4.01 (2.12–7.57)	2.71 (1.34–5.48)	2.21 (1.03–4.77)
Metronidazole	5.59 (2.69–11.63)	2.48 (1.04–5.94)	1.38 (0.55–3.45)
SDD	7.02 (3.76–13.02)	4.35 (1.46–12.99)	10.24 (2.84–36.85)
Vancomycin	2.85 (1.42–5.72)	1.41 (0.62–3.24)	0.96 (0.40–2.28)
Macrolides	3.38 (1.63–7.02)	1.68 (0.73–3.89)	1.11 (0.45–2.73)
Clindamycin	0.83 (0.28–2.51)	0.74 (0.23–2.39)	NA
Carbapenems	1.64 (0.70–3.83)	0.84 (0.32–2.21)	NA
Aminoglycoside	1.62 (0.61–4.34)	2.09 (0.76–5.81)	NA

The univariate and multivariable analysis was done by conditional logistic regression.

^#^ Adjusted for length of stay (admission day-index date), sex, Charlson comorbidity index, and stay at ICU

^##^ Adjusted for length of stay (admission day-index date), sex, Charlson comorbidity index, stay at ICU, and the use of antibiotic classes associated in univariate analysis (cephalosporins 2^nd^ and 3^rd^ generation without SDD, penicillins, quinolones, metronidazole, SDD, vancomycin, and macrolides were separately entered into the multivariable model).

SDD: selective decontamination of the digestive tract; NA: not applicable

#### CDI due to ribotype 027 versus CDI due to other ribotypes

To distinguish between general CDI risk factors and factors that were specifically related to this 027 outbreak, CDI ribotype 027 cases were also compared to patients with CDI due to other ribotypes. The main risk factors associated with CDI ribotype 027 found in the comparison with non-CDI patients: use of antibiotics, admission to the ICU, and hospital LOS, were analyzed in a second multivariable model comparing patients with CDI ribotype 027 to control patients with CDI due to other ribotypes. In this model, admission to the ICU (OR 5.5, 95% CI 2.1–14.3), and hospital LOS (OR 1.9, 95% C.I. 1.3–2.8) remained significantly associated with the development of CDI ribotype 027. After adjustment for ICU admission, and LOS, the use of SDD was also associated with the development of CDI ribotype 027 (OR 8.0, 95% C.I. 1.3–48.2).

### Patients staying at ICU prior to the index date

Because SDD, as defined in this study, is only used at the ICU, we performed an analysis restricted to those patients who had stayed at the ICU (35 cases; 51 non-CDI controls) prior to the index date. In the univariate model (Table 4), cases were more likely to have used SDD in the previous 90 days compared to controls, and had a longer hospital and ICU LOS. There was no association with sex, Charlson’s index, APACHE IV score, use of PPIs, number of ward transfers, recent admission to the hospital, and admission to a ward with an isolated CDI patient. Because of possible confounding by LOS, number of ward transfers, and severity of disease, we analyzed use of SDD, hospital LOS, ICU LOS, number of ward transfers, and APACHE IV score in a multivariable model, which showed similar point estimates for SDD and ICU LOS, with slightly wider confidence intervals (Table 5).

**Table 4:** Patient characteristics and crude odds ratios of potential risk factors for the development of CDI ribotype 027 in participants who had stayed at the ICU.

Risk factors		CDI patients (N = 35)		Non-CDI control patients (N = 51)		Crude OR (95% CI)
		N	(%)	N	(%)	
Age (mean, ± SD)		60.7	(±20.4)	63.6	(±15.1)	0.99 (0.97–1.02)
Male		24	(69)	36	(71)	0.91 (0.36–2.31)
Charlson comorbidity index (mean ± SD)		1.9	(±1.9)	1.6	(±1.3)	1.11 (0.84–1.47)
APACHE IV score (mean ± SD)		66.0	(±25.1)	79.4	(28.6)	0.98 (0.97–1.00)
Any antibiotic therapy (-90 days)		35	(100)	49	(96)	NA
Antibiotic classes						
	Cephalosporins 2^nd^ and 3^rd^ generation without SDD	1	(3)	3	(6)	0.47 (0.05–4.72)
	Penicillins	15	(43)	17	(33)	1.50 (0.62–3.64)
	Quinolones	12	(34)	20	(39)	0.81 (0.33–1.98)
	Metronidazole	11	(31)	11	(22)	1.67 (0.63–4.43)
	SDD	34	(97)	42	(82)	7.29 (0.88–60.39)
	Clindamycin	1	(3)	2	(4)	0.72 (0.06–8.27)
	Vancomycin	8	(23)	9	(18)	1.38 (0.48–4.02)
	Carbapenems	4	(11)	7	(14)	0.81 (0.21–3.01)
	Aminoglycosides	1	(3)	3	(6)	0.47 (0.05–4.72)
	Macrolides	9	(26)	11	(22)	1.42 (0.51–3.96)
Proton pump inhibitors (-90 days)		22	(63)	26	(51)	1.63 (0.68–3.92)
Number of ward transfers						
	0	1	(3)	6	(12)	Reference
	1–2	17	(49)	27	(53)	3.77 (0.42–34.17)
	≥ 3	17	(49)	18	(35)	5.67 (0.62–52.09)
Hospital LOS before index date (mean ±SD)		27.5	(±24.4)	18.1	(±19.2)	1.61 (1.03–2.51)
ICU LOS before index date (mean ± SD)		16.0	(±15.9)	10.3	(±13.1)	1.74 (1.14–2.67)
Previous admission (-90 days)		6	(17)	8	(16)	1.11 (0.35–3.54)
Admission to the same ward as an isolated CDI patient		24	(69)	17	(73)	0.83 (0.32–2.12)

The univariate analysis was done by logistic regression; APACHE: Acute Physiology and Chronic Health Evaluation; SDD: selective decontamination of the digestive tract; LOS: length of stay; NA: not applicable

**Table 5:** Adjusted odds ratios of risk factors for CDI ribotype 027 in participants who had stayed at the ICU.

Risk factors		Adjusted^#^ OR (95% CI)
APACHE IV score (mean)		0.97 (0.95–0.99)
SDD		7.90 (0.71–88.02)
Hospital LOS before index date (mean)		1.25 (0.59–2.68)
ICU LOS before index date (mean)		1.53 (0.75–3.10)
Number of ward transfers		
	0	Reference
	1–2	3.09 (0.31–31.23)
	≥ 3	3.18 (0.27–37.26)

The multivariable analysis was done by logistic regression.

^#^ Model adjusted for ICU LOS, hospital LOS, use of SDD, number of ward transfers, and APACHE IV score

APACHE: Acute Physiology and Chronic Health Evaluation; SDD: selective decontamination of the digestive tract; LOS: length of stay; NA: not applicable

Only seven of the 70 patients with CDI due to other ribotypes had been admitted to the ICU during the outbreak period, which made it impossible to perform a restricted analysis for this group.

### Clinical course and outcome

Of 79 cases, 14 patients died within 30 days, accounting for 18% mortality, compared to 9% (27 of 302 controls with known follow up) in the non-CDI control patients, and 14% in the control group of patients with CDI due to other ribotypes. The mortality among patients with CDI was still significantly higher than among non-CDI controls after adjustment for age, sex and Charlson’s index (RR 2.2, 95% CI 1.1–4.4). Of the case patients, 21 patients (27%) were admitted to the ICU or MCU after the index date, compared to 51 of 316 control patients (16%). Case patients had a mean total hospital LOS of 47 days (SD ± 35), control patients of 27 days (SD ± 26). One patient with CDI underwent colectomy, because of severe pseudomembranous colitis. Recurrent CDI (defined as a new episode of diarrhea with a positive stool for *C*. *difficile* after discontinuation of antibiotic therapy for CDI within three months from the last episode of CDI) occurred in 29 patients (37%).

## Discussion

In this case control study of the largest described outbreak of CDI in the Netherlands we found an association between CDI due to ribotype 027 and use of antibiotics, prior admission to the ICU, recent admission to the hospital, and a longer hospital LOS prior to index date, while admission to a ward where a case of CDI was nursed in contact isolation appeared as a protective factor that reduced the risk of CDI by 75%. Except for admission to the ICU, or to the same ward as an isolated case, other hospital-associated factors did not contribute to the spread of the outbreak strain. When we compared cases with CDI ribotype 027 to control patients with CDI due to other ribotypes, admission to the ICU, and hospital LOS remained associated with CDI ribotype 027. In this comparison, the use of SDD was also significantly associated with CDI ribotype 027. For those patients who had been admitted to the ICU prior to the index date, use of SDD, hospital LOS, and ICU LOS were strongly associated with CDI in univariate analysis. In the multivariable analysis, the association between SDD use and CDI retained the same point estimate, with slightly wider confidence interval. The mortality rate among patients with CDI was 10% higher than among non-CDI control patients, and 4% higher than among control patients with CDI due to other ribotypes.

The outbreak involved nearly 80 patients before coming to an end after the implementation of several control measures: reinforcement of infection control measures (use of aprons and gloves, appropriate hand hygiene, isolation in single rooms, and the introduction of hydrogen peroxide as disinfectant), optimization of CDI diagnosis and CDI treatment, and antibiotic stewardship (including restriction of quinolone use in all departments).

A strength of our study is its large size and the completeness of data, that enabled us to adjust for important clinical variables. Matching cases and controls on age, attending specialty, and ward within 48 hours of CDI diagnosis, ensured us that these patients originated from a setting with comparable CDI pressure. We matched for attending specialty and ward as a proxy for underlying disease; that Charlson’s index was comparable for cases and controls showed that matching was successful. In the analysis we adjusted for sex, Charlson’s index, hospital LOS, and admission to the ICU. In an analysis restricted to those patients who had stayed at the ICU, we adjusted for LOS in the hospital and LOS in the ICU, and APACHE IV score. We therefore consider that we provided a well-substantiated estimate of the risk factors for the development of CDI due to ribotype 027 in this outbreak. By comparing CDI ribotype 027 cases to control patients with CDI due to other ribotypes in a second model, we were able to distinguish general risk factors for CDI from factors specifically associated with the emergence of this predominant strain.

Our study has limitations. Firstly, we used data from an electronic hospital database, with the potential of misclassification. Secondly, we recorded the in-hospital use of antibiotics within 90 days before the index date, but could not track the use of antibiotics outside the hospital. Thirdly, the second control group consisting of patients with CDI due to other ribotypes was not matched for calendar time at the individual patient level, but only at group level. Fourthly, the ICU receives and discharges the most severely ill patients, who are therefore most at risk for acquiring CDI. We did adjust for APACHE IV score, and LOS in the ICU, but we cannot rule out residual confounding due to underlying diseases or disease severity in the development of CDI. Still, it should be noted that most studies on ICU patients consider that the APACHE score is an adequate variable to adjust for severity of disease in multivariable models [13]. Finally, the study was not originally designed to address the impact of the ICU nor the impact of SDD. The sample size of the restricted analysis was limited, this affects the precision of the estimation of the ORs and their 95% CI.

The most important risk factor for CDI is the use of antibiotics [4]. We have confirmed this finding: 95% of the cases during this outbreak had used antibiotics in the 90 days prior to the diagnosis of CDI. This implies that antibiotics are almost an essential prerequisite to develop CDI, even in an outbreak setting. Several studies have suggested that PPI may be a risk factor for CDI, but other studies could not confirm this [14]. We found no association with PPI use in the multivariable analysis, but it should be noted that nearly two thirds of control patients used PPIs. The finding that stay on a ward where cases were nursed in isolation was protective seems counterintuitive. However, this may possibly be due to extra vigilance of hospital staff, patients and visitors, intensified hygienic measures, and cleaning of environment on that particular ward in this epidemic setting.

Almost half of case patients, but only one sixth of non-CDI control patients, and one tenth of the non-027 CDI patients had been admitted to the ICU prior to the index date. With regard to the in-hospital traffic of patients, the ICU generally takes a central position. They receive and discharge our most severely ill patients to all the different wards of the hospital. The traffic to and from the ICU could therefore play a major role in inducing or maintaining a hospital-wide outbreak. That admission to the ICU remained significantly associated with CDI ribotype 027 when we compared the cases to control patients with CDI due to other ribotypes supports this hypothesis. Previous admission to an ICU has been recognized as a risk factor for CDI in endemic situations [15], and it has been suggested previously that ICU populations demand additional resources and attention from an infective prevention perspective to control the hospital-wide spread of *C*. *difficile* [16].

Analysis of those patients who had been admitted to the ICU prior to the index date, showed that the use of SDD, and a longer LOS in the ICU were both associated with the development of CDI in the univariate analysis. SDD is given to all patients with an expected ICU stay of more than two days and can therefore be an indicator of more serious illness, resulting in longer ICU stay. Numbers were too small to disentangle the effect of SDD and the effect of a prolonged LOS in the ICU. This applies even more for the comparison between cases with CDI ribotype 027, and controls with CDI due to other strains, an analysis based on smaller numbers. Therefore, we have to scrutinize the biological plausibility of both risk factors. SDD is aimed at preventing or eradicating the oropharyngeal and intestinal carriage of potentially pathogenic microorganism in critically ill patients. It is used in all ICUs in the Netherlands, and in a small number of ICUs in some countries in Europe. After exposure to oral antibiotics, a rapid decline in fecal microbiota diversity is common and may last for months [17,18]. Since the imbalance in gut flora plays a key role in the pathophysiology of CDI, and a larger amount of antibiotics, more different classes of antibiotics, and a longer duration of antibiotic therapy are all associated with an increased risk of CDI, this increased risk for the development of CDI in patients that have received SDD appears a biologically plausible finding [19,20]. There is debate about the safety of SDD in relation to the development of resistance [21]. Our findings of the possibility of an increased risk of developing CDI due to *C*. *difficile* ribotype 027 add to this debate.

A longer duration of hospital stay is a well-defined risk factor for the development of CDI: it increases the chances of exposure to *C*. *difficile* spores either by indirect contact with a contaminated surface or by direct contact with an infected person [5,22,23]. However, longer LOS is most likely a marker of severity of disease or increased antibiotic administration. Although in our study the APACHE IV score was comparable between cases and controls, patients hospitalized in ICUs represent a highly vulnerable population due to severe underlying disease, extensive use of antibiotics, including SDD, exposure to invasive procedures, and frequent contacts with healthcare workers that increase potential exposure to *C*. *difficile* spores at the ICU. Presumably, the combination of all these factors contributed to the high incidence of CDI ribotype 027 in patients who had been admitted to the ICU prior to CDI diagnosis. Of note, most of these case patients did not develop CDI while staying on the ICU, but only after discharge from the ICU, which supports a possible role of a long-lasting disturbance of the gut microbiota in the development of CDI.

In conclusion, in this outbreak setting, caused by a single clone of *C*. *difficile* ribotype 027, as confirmed by WGS, antibiotic use appeared as a prerequisite for infection with the outbreak strain. Stay at the ICU, and use of SDD, which is related to LOS in the ICU, were the main factors associated with the development of CDI ribotype 027 during this outbreak. Our study underlines that during a period of high CDI incidence or during a CDI outbreak in a hospital, any use of antibiotics, including the use of SDD, could be a contributing factor in maintaining the outbreak. This also stresses the need for stringent infection control preventive measures at the ICU during an outbreak.

## References

[1] Ghose C. Clostridium difficile infection in the twenty-first century. Emerg Microbes Infect 2013 9;2(9):e62 10.1038/emi.2013.62 26038491 PMC3820989

[2] Loo VG, Poirier L, Miller MA, Oughton M, Libman MD, Michaud S, et al A predominantly clonal multi-institutional outbreak of Clostridium difficile-associated diarrhea with high morbidity and mortality. N Engl J Med 2005 12 8;353(23):2442–9. 16322602 10.1056/NEJMoa051639

[3] Miller M, Gravel D, Mulvey M, Taylor G, Boyd D, Simor A, et al Health care-associated Clostridium difficile infection in Canada: patient age and infecting strain type are highly predictive of severe outcome and mortality. Clin Infect Dis 2010 1 15;50(2):194–201. 10.1086/649213 20025526

[4] Leffler DA, Lamont JT. Clostridium difficile infection. N Engl J Med 2015 4 16;372(16):1539–48. 10.1056/NEJMra1403772 25875259

[5] Ananthakrishnan AN. Clostridium difficile infection: epidemiology, risk factors and management. Nat Rev Gastroenterol Hepatol 2011 1;8(1):17–26. 10.1038/nrgastro.2010.190 21119612

[6] Stevens VW, Khader K, Nelson RE, Jones M, Rubin MA, Brown KA, et al Excess Length of Stay Attributable to Clostridium difficile Infection (CDI) in the Acute Care Setting: A Multistate Model. Infect Control Hosp Epidemiol 2015 9;36(9):1024–30. 10.1017/ice.2015.132 26006153

[7] van Prehn J, Vandenbroucke-Grauls C, van Beurden Y, van Houdt R, Vainio S, Ang C. Diagnostic yield of repeat sampling with immunoassay, real-time PCR, and toxigenic culture for the detection of toxigenic Clostridium difficile in an epidemic and a non-epidemic setting. Eur J Clin Microbiol Infect Dis 2015 9 16. 10.1007/s10096-015-2484-9 PMC4655006 26377204

[8] Bruijnesteijn van Coppenraet LES, Savelkoul PHM, Buffing N, van der Bijl MW, Woudenberg J, Lindeboom JA, et al Amplified fragment length polymorphism analysis of human clinical isolates of Mycobacterium haemophilum from different continents. Clin Microbiol Infect 2009 10;15(10):924–30. 10.1111/j.1469-0691.2009.02798.x 19659689

[9] Bentley DR, Balasubramanian S, Swerdlow HP, Smith GP, Milton J, Brown CG, et al Accurate whole human genome sequencing using reversible terminator chemistry. Nature 2008 11 6;456(7218):53–9. 10.1038/nature07517 18987734 PMC2581791

[10] Charlson ME, Pompei P, Ales KL, MacKenzie CR. A new method of classifying prognostic comorbidity in longitudinal studies: development and validation. J Chronic Dis 1987;40(5):373–83. 3558716 10.1016/0021-9681(87)90171-8

[11] Zimmerman JE, Kramer AA, McNair DS, Malila FM. Acute Physiology and Chronic Health Evaluation (APACHE) IV: hospital mortality assessment for today's critically ill patients. Crit Care Med 2006 5;34(5):1297–310. 16540951 10.1097/01.CCM.0000215112.84523.F0

[12] Knetsch CW, Connor TR, Mutreja A, van Dorp SM, Sanders IM, Browne HP, et al Whole genome sequencing reveals potential spread of Clostridium difficile between humans and farm animals in the Netherlands, 2002 to 2011. Euro Surveill 2014;19(45):20954 25411691 10.2807/1560-7917.es2014.19.45.20954 PMC4518193

[13] Oostdijk EAN, Kesecioglu J, Schultz MJ, Visser CE, de Jonge E, van Essen EHR, et al Effects of decontamination of the oropharynx and intestinal tract on antibiotic resistance in ICUs: a randomized clinical trial. JAMA 2014 10 8;312(14):1429–37. 10.1001/jama.2014.7247 25271544

[14] Janarthanan S, Ditah I, Adler DG, Ehrinpreis MN. Clostridium difficile-associated diarrhea and proton pump inhibitor therapy: a meta-analysis. Am J Gastroenterol 2012 7;107(7):1001–10. 10.1038/ajg.2012.179 22710578

[15] Hensgens MPM, Goorhuis A, van Kinschot CMJ, Crobach MJT, Harmanus C, Kuijper EJ. Clostridium difficile infection in an endemic setting in the Netherlands. Eur J Clin Microbiol Infect Dis 2011 4;30(4):587–93. 10.1007/s10096-010-1127-4 21194003 PMC3052466

[16] Lofgren ET, Cole SR, Weber DJ, Anderson DJ, Moehring RW. Hospital-acquired Clostridium difficile infections: estimating all-cause mortality and length of stay. Epidemiology 2014 7;25(4):570–5. 10.1097/EDE.0000000000000119 24815305 PMC4224274

[17] Jernberg C, Lofmark S, Edlund C, Jansson JK. Long-term impacts of antibiotic exposure on the human intestinal microbiota. Microbiology 2010 11;156(Pt 11):3216–23. 10.1099/mic.0.040618-0 20705661

[18] Dethlefsen L, Huse S, Sogin ML, Relman DA. The pervasive effects of an antibiotic on the human gut microbiota, as revealed by deep 16S rRNA sequencing. PLoS Biol 2008 11 18;6(11):e280 10.1371/journal.pbio.0060280 19018661 PMC2586385

[19] Hensgens MPM, Goorhuis A, Dekkers OM, Kuijper EJ. Time interval of increased risk for Clostridium difficile infection after exposure to antibiotics. J Antimicrob Chemother 2012 3;67(3):742–8. 10.1093/jac/dkr508 22146873

[20] Stevens V, Dumyati G, Fine LS, Fisher SG, van Wijngaarden E. Cumulative antibiotic exposures over time and the risk of Clostridium difficile infection. Clin Infect Dis 2011 7 1;53(1):42–8. 10.1093/cid/cir301 21653301

[21] Daneman N, Sarwar S, Fowler RA, Cuthbertson BH. Effect of selective decontamination on antimicrobial resistance in intensive care units: a systematic review and meta-analysis. Lancet Infect Dis 2013 4;13(4):328–41. 10.1016/S1473-3099(12)70322-5 23352693

[22] Archibald LK, Banerjee SN, Jarvis WR. Secular trends in hospital-acquired Clostridium difficile disease in the United States, 1987–2001. J Infect Dis 2004 5 1;189(9):1585–9. 15116293 10.1086/383045

[23] Lawrence SJ, Puzniak LA, Shadel BN, Gillespie KN, Kollef MH, Mundy LM. Clostridium difficile in the intensive care unit: epidemiology, costs, and colonization pressure. Infect Control Hosp Epidemiol 2007 2;28(2):123–30. 17265392 10.1086/511793

